# Supplementary material for: Coverage evaluation surveys following soil-transmitted helminthiasis and schistosomiasis mass drug administration in Wolaita Zone of Ethiopia—The Geshiyaro project
Source: PLoS One. 2021 Dec 21;16(12):e0260722. doi: 10.1371/journal.pone.0260722 (PMC8691621; doi:10.1371/journal.pone.0260722)
Supplement: S1 Appendix — (DOCX) [file pone.0260722.s001.docx]

S1.1 Appendix: Parameter values used for sample size calculation

| **Woreda** | **Parameter values for sample size calculation** | | | | | Sample Size |
| --- | --- | --- | --- | --- | --- | --- |
|  | Coverage(*p*) | Design Effect (*DEFF*) | Margin of error (δ) | Critical Value (Z_α/2_) | Non response rate (*r*) |  |
| Abela Abaya | 0.68 | 4 | 0.05 | 1.96 | 0.15 | 1574 |
| Bolosso Sore | 0.78 |  |  |  |  | 1241 |
| Duguna Fango | 0.75 |  |  |  |  | 1356 |
| Sodo Zuria | 0.75 |  |  |  |  | 1356 |
| Tebela Town | 0.75 |  |  |  |  | 1356 |
| Humbo Woreda | 0.75 |  |  |  |  | 1356 |
| Hobicha | 0.75 |  |  |  |  | 1356 |
| Sodo Town | 0.75 |  |  |  |  | 1356 |
| Bayra Koysha | 0.75 |  |  |  |  | 1356 |
| Kawo Kysha | 0.75 |  |  |  |  | 1356 |
| Gesuba Town | 0.75 |  |  |  |  | 1356 |
| Offa | 0.75 |  |  |  |  | 1356 |
| Kindo Koysha | 0.75 |  |  |  |  | 1356 |

S1.2 Appendix: Household Questionnaire, adapted from the WHO

| **Section** | **Question** | **Possible answers** | **Notes** |
| --- | --- | --- | --- |
| **INTERVIEWER** | Interviewer name | Select from drop down list or enter manually |  |
| **Woreda** | Please select the correct woreda | Select from drop down list |  |
| **KEBELE** | Please select the correct kebele | Select from drop down list |  |
| **Subunit/EA** | What is the name of the enumeration area? | Select from drop down list |  |
| **GPS** | Record GPS location of a compound |  |  |
| **Number of interviewee** | Number of individuals **in the household (age ≥1 years)** |  |  |
| **CONSENT AND ENROLMENT** | Does the individual consent to participate in the coverage survey? | Yes/No |  |
|  | Have you lived in this kebele for the past 3 months? | Yes/No | To determine if an individual was resident in the kebele during the MDA round |
| **COVERAGE SURVEY (all eligible individuals in the household)** | Name | Name of a participant |  |
|  | Age | *Enter number* |  |
|  | Sex | Male/Female |  |
|  | Did this child attend school in the last year? | Yes/No | For school-aged children only |
|  | Were you offered Albendazole? | Yes/No/DK | Hint: Show the individual a bottle of ALB tablets |
|  | Why were not you offered albendazole? | 1=Underage, 2=Pregnant, 3= Breast feeding, 4=Too sick, 5=Absent, 6=Not heard about program, 7=Drugs finished/runout, 8=HEW didn’t come, 88=Other | Relevant when “No” is selected for Were you offered albendazole? |
|  | Reason_other | What is the reason not offered? | Relevant when “other” is selected for question: Why didn’t you offered albendazole? above |
|  | Did you swallow this tablet/albendazole? | Yes/No/DK |  |
|  | Why didn’t you swallow albendazole? | 1=Fear of side effects, 2=Bad test, 3=Not sick, 4= Not enough information given, 88=Other | Relevant when “No” is selected for question: Did you swallow this tablet/albendazole? above |
|  | Were you offered praziquantel? | Yes/No/DK | Hint: Show the individual a bottle of PZQ tablets and dose pole |
|  | Why were not you offered praziquantel? | 1=Underage, 2=Pregnant, 3= Breast feeding, 4=Too sick, 5=Absent, 6=Not heard about program, 7=Drugs finished/runout, 8=HEW didn’t come, 88=Other | Relevant when “No” is selected for question “Were you offered praziquantel”? above |
|  | Reason_other_PZQ | What is the reason not offered? | Relevant when “other” is selected for question: Why didn’t you offered praziquantel? above |
|  | Did you swallow praziquantel? | Yes/No/DK |  |
|  | Why did you not swallow praziquantel? | 1=Fear of side effects, 2=Bad test, 3=Not sick, 4= Not enough information given, 88=Other | Relevant when “no” is selected for question” Did you swallow praziquantel?” above |
|  | Where did you take the treatment? | 1=In the community through 1=In the community through HEW,  2= At school, 3=At a health facility,  4=At home,  88=Other |  |
|  | Did you experience any side effects after taking treatment? | Yes/No |  |
|  | What side effects did you experience? | 1=Nausea; 2=Vomiting; 3=Headache; 4=Dizziness; 5=Fainting; 6=Fatigue; 88=Other | Relevant when “Yes” is answered for question “Did you experience any side effects after taking treatment?” above |
|  | How did you hear about the MDA? | 1=HEW, 2=Village leader, 3=Religious leader, 4=Posters, 5=Town crier, 6=Other (can be multiple choice selection) | Do not list the answers to the individual |
|  | Next time the MDA is carried out, in what location would you like the MDA to take place? | 1=In the community through HEW (Fixed-point MDA)  2= At home (House-to-House MDA) 3=School  4=Health Centre (Test & Treat, not MDA)  88=Other |  |
|  | What was your motivation for taking part in the MDA? | 1=Fear of disease(s), 2=To treat disease(s), 3=Because it was given free, 4=Useful information from community drug distributors (CDD), 88=Other |  |

**NB**: The survey team should bring examples of each drug to show the participants at the time of questioning to help with recall.

S1.3 Appendix: Validation interpretation, adapted from WHO coverage evaluation survey

| **Conclusion** | **Validation Interpretation** |
| --- | --- |
| If survey coverage validates reported coverage | The reported coverage is contained within the 95% confidence interval around the survey coverage. This means the reported coverage can be considered be "validated" in that IU; no action or improvements are required to the reporting system. |
| If survey and reported coverage are similar | The reported coverage is outside the 95% confidence interval around the survey coverage but is still within +/- 10 percentage points of the survey coverage, which suggests the reporting system is working well; no action or improvements are required to the reporting system. |
| If survey coverage is less than reported coverage | The reported coverage is between +/- 10 to 25 percentage points different from the survey coverage. This suggests there could be a problem with the reporting system and action may be required if resources permit. |
| If survey coverage is much less than reported coverage | The reported coverage is at least +/- 25 percentage points different from the survey coverage. This suggests that there is a real problem with the reported coverage and follow-up action to improve the reporting system in the IU is required. |
