# Supplementary material for: Coverage evaluation surveys following soil-transmitted helminthiasis and schistosomiasis mass drug administration in Wolaita Zone of Ethiopia—The Geshiyaro project
Source: PLoS One. 2021 Dec 21;16(12):e0260722. doi: 10.1371/journal.pone.0260722 (PMC8691621; doi:10.1371/journal.pone.0260722)
Supplement: Attachment — Submitted filename: LRLPONE-D-21-27754_reviewer.pdf [file pone.0260722.s003.pdf]

PLOS ONE

Coverage evaluation surveys following soil-transmitted helminthiasis and schistosomiasis mass drug administration in Wolaita Zone of Ethiopia- The Geshiyaro project

--Manuscript Draft--

**Manuscript Number:**	PONE-D-21-27754
**Article Type:**	Research Article
**Full Title:**	Coverage evaluation surveys following soil-transmitted helminthiasis and schistosomiasis mass drug administration in Wolaita Zone of Ethiopia- The Geshiyaro project
**Short Title:**	Coverage evaluation surveys in Wolaita Zone
**Corresponding Author:**	Ewnetu Firdawek Liyew, PhD student<br>Ethiopian Public Health Institute<br>Addis Ababa, ETHIOPIA
**Keywords:**	Coverage evaluation surveys; chemotherapy; albendazole; praziquantel; treatment coverage; survey coverage; administrative reported coverage; soil-transmitted helminthiasis; schistosomiasis; mass drug administration
**Abstract:**	Introduction: The goal of the Geshiyaro project is to break transmission of soil-transmitted helminthiasis and schistosomiasis in the Wolaita Zone of Ethiopia through preventive chemotherapy with albendazole (ALB) and praziquantel (PZQ), with the goal of 90% treatment coverage. To compare reported administrative coverage, and ensure that the Geshiyaro threshold had been met, post-treatment coverage evaluation surveys (CES) were conducted to monitor the success of each round.<br>Methods: A community-based cross-sectional study was conducted in 13 woredas (districts) of the Wolaita Zone. All eligible individuals from the selected households were invited for an interview. The study design, sample size, analysis and report writing was done according to the World Health Organization CES guidelines for preventive chemotherapy.<br>Results: The study interviewed a total of 3,568 households and 18,875 individuals across 13 woredas in the Wolaita Zone. Overall, the survey coverage across all studied woredas was 81.5% (95% CI; 80.9 – 82.0) for both ALB and PZQ. Reported administrative coverage across all studied woredas was higher than survey coverage, 92.7% and 91.2% for ALB and PZQ respectively. Significant portion of the beneficiaries (17.6%) were not offered the drugs, and the main reason for not achieving the target coverage included beneficiaries absenteeism during Mass Drug Administration (MDA) (6.6% ALB, 6.8% PZQ), drug distributors failed to go to all households (4.7% ALB, 4.8% PZQ), and the beneficiaries unable to hear about the program (1.3% ALB, 1.7% PZQ).<br>Conclusion: It is important to take programmatic actions during the next MDA campaign to achieve the minimum Geshiyaro coverage threshold. Adequate training and supervision on reporting and tallying should be given to the drug distributers, supervisors and staff of the Ministry of Health (MoH). It is important to strengthen the social mobilization, motivation of drug distributers, and enhancing the engagement of MoH staff during the future MDA campaign.
**Order of Authors:**	Ewnetu Firdawek Liyew, PhD student
	Melkie Chernet
	Habtamu Belay
	Rosie Maddren
	Toby Landeryou
	Suprabhath Kalahasti
	Alison K Ower
	Kalkidan Mekete

Powered by Editorial Manager® and ProduXion Manager® from Aries Systems Corporation

	Anna E. Phillips
	Ufaysa Anjulo
	Tujuba Endrias
	Adugna Tamiru
	Bokretsion Gidey
	Zelalem Mehari
	Birhan Mengistu
	Getachew Tollera
	Geremew Tasew
**Additional Information:**	
**Question**	**Response**
**Financial Disclosure**<br><br>Enter a financial disclosure statement that describes the sources of funding for the work included in this submission. Review the submission guidelines for detailed requirements. View published research articles from *PLOS ONE* for specific examples.<br><br>This statement is required for submission and **will appear in the published article** if the submission is accepted. Please make sure it is accurate.<br><br>**Unfunded studies**<br>Enter: *The author(s) received no specific funding for this work.*<br><br>**Funded studies**<br>Enter a statement with the following details:<br>• Initials of the authors who received each award<br>• Grant numbers awarded to each author<br>• The full name of each funder<br>• URL of each funder website<br>• Did the sponsors or funders play any role in the study design, data collection and analysis, decision to publish, or preparation of the manuscript?<br>• **NO** - Include this sentence at the end of your statement: *The funders had no role in study design, data collection and analysis, decision to publish, or preparation of the manuscript.*<br>• **YES** - Specify the role(s) played.<br><br>* typeset	This research was funded by the Child Investment Fund Foundation (CIFF), UK under grant number R-1805-02741. The funders had no role in the design of the study, collection, analysis and interpretation of the data. The finding and conclusion of the study reflect the view of the<br>Authors only.

**Competing Interests**<br><br>Use the instructions below to enter a competing interest statement for this submission. On behalf of all authors, disclose any competing interests that could be perceived to bias this work—acknowledging all financial support and any other relevant financial or non-financial competing interests.<br><br>This statement is required for submission and **will appear in the published article** if the submission is accepted. Please make sure it is accurate and that any funding sources listed in your Funding Information later in the submission form are also declared in your Financial Disclosure statement.<br><br>View published research articles from *PLOS ONE* for specific examples.<br><br>**NO authors have competing interests**<br><br>Enter: *The authors have declared that no competing interests exist.*<br><br>**Authors with competing interests**<br><br>Enter competing interest details beginning with this statement:<br><br>*I have read the journal's policy and the authors of this manuscript have the following competing interests: [insert competing interests here]*<br><br>* typeset	I have read the journal's policy and the authors of this manuscript have the following competing interests: ZM is the Manager, Evidence Measurement and Evaluation at the Children's Investment Fund Foundation. BM is the Program Manager at the Children's Investment Fund Foundation, the project's funder
**Ethics Statement**<br><br>Enter an ethics statement for this submission. This statement is required if the study involved:<br><br>• Human participants<br>• Human specimens or tissue<br>• Vertebrate animals or cephalopods	The study was approved by Institutional Review Board (IRB) at the Scientific and Ethical Review Office of the Ethiopian Public Health Institute. Letter of support and explanation about the purpose of the study was provided to all concerned bodies. Before conducting any interview, verbal consent was taken from the head of the household or an adult after providing all necessary information about the study in the local language that every participant can understand. Assent for young children (

• Vertebrate embryos or tissues<br>• Field research<br><br>Write "N/A" if the submission does not require an ethics statement.<br><br>General guidance is provided below. Consult the submission guidelines for detailed instructions. **Make sure that all information entered here is included in the Methods section of the manuscript.**	

Powered by Editorial Manager® and ProduXion Manager® from Aries Systems Corporation

**Format for specific study types**<br><br>**Human Subject Research (involving human participants and/or tissue)**<br>• Give the name of the institutional review board or ethics committee that approved the study<br>• Include the approval number and/or a statement indicating approval of this research<br>• Indicate the form of consent obtained (written/oral) or the reason that consent was not obtained (e.g. the data were analyzed anonymously)<br><br>**Animal Research (involving vertebrate animals, embryos or tissues)**<br>• Provide the name of the Institutional Animal Care and Use Committee (IACUC) or other relevant ethics board that reviewed the study protocol, and indicate whether they approved this research or granted a formal waiver of ethical approval<br>• Include an approval number if one was obtained<br>• If the study involved *non-human primates*, add *additional details* about animal welfare and steps taken to ameliorate suffering<br>• If anesthesia, euthanasia, or any kind of animal sacrifice is part of the study, include briefly which substances and/or methods were applied<br><br>**Field Research**<br><br>Include the following details if this study involves the collection of plant, animal, or other materials from a natural setting:<br>• Field permit number<br>• Name of the institution or relevant body that granted permission	
**Data Availability**<br><br>Authors are required to make all data underlying the findings described fully available, without restriction, and from the time of publication. PLOS allows rare exceptions to address legal and ethical concerns. See the PLOS Data Policy and FAQ for detailed information.	Yes - all data are fully available without restriction

A Data Availability Statement describing where the data can be found is required at submission. Your answers to this question constitute the Data Availability Statement and **will be published in the article**, if accepted.<br><br>**Important:** Stating 'data available on request from the author' is not sufficient. If your data are only available upon request, select 'No' for the first question and explain your exceptional situation in the text box.<br><br>Do the authors confirm that all data underlying the findings described in their manuscript are fully available without restriction?	
**Describe where the data may be found in full sentences. If you are copying our sample text, replace any instances of XXX with the appropriate details.**<br><br>• If the data are **held or will be held in a public repository**, include URLs, accession numbers or DOIs. If this information will only be available after acceptance, indicate this by ticking the box below. For example: *All XXX files are available from the XXX database (accession number(s) XXX, XXX.*).<br>• If the data are all contained **within the manuscript and/or Supporting Information files**, enter the following: *All relevant data are within the manuscript and its Supporting Information files.*<br>• If neither of these applies but you are able to provide **details of access elsewhere**, with or without limitations, please do so. For example:<br><br>*Data cannot be shared publicly because of [XXX]. Data are available from the XXX Institutional Data Access / Ethics Committee (contact via XXX) for researchers who meet the criteria for access to confidential data.*<br><br>*The data underlying the results presented in the study are available from (include the name of the third party*	De-identified individual participant data will be made available on publication, subject to request, to members of the scientific and medical communities for non-commercial use only

and contact information or URL).<br>• This text is appropriate if the data are owned by a third party and authors do not have permission to share the data.<br><br>* typeset	
Additional data availability information:	

Manuscript

Click here to access/download;Manuscript;Manuscript PLOS ONE 26Aug2021.docx

Coverage evaluation surveys following soil-transmitted helminthiasis and schistosomiasis mass drug administration in Wolaita Zone of Ethiopia- The Geshiyaro project

Ewnetu Firdawek*[1] , Melkie Chernet[1] , Habtamu Belay [1] , Rosie Maddren[2,3], Toby Landeryou[2,3], Kalahasti, Suprabhath[2], Alison K Ower[2,3], Kalkidan Mekete[1], Anna E. Phillips[2,3], Ufaysa Anjulo[4], Tujuba Endrias[1], Adugna Tamiru[5] , Bokretsion Gidey[1], Zelalem Mehari[6] , Birhan Mengistu[6], Getachew Tollera [1], Geremew Tasew[1]

[1]Ethiopian Public Health Institute, Addis Ababa, Ethiopia

[2]Department of Infectious Disease Epidemiology, School of Public Health, Faculty of Medicine, Imperial College London, London, W2 1PG, UK.

[3]London Centre for Neglected Tropical Disease Research, London, UK.

[4]Ministry of Health, Wolaita, Ethiopia

[5]Ministry of Health, Addis Ababa, Ethiopia

[6]Children's Investment Fund Foundation, London, WS1 2FT, UK

*Correspondence: ewnetuliyew@gmail.com

Authors email addresses:

EF: ewnetuliyew@gmail.com

MC: melkie.chernet@yahoo.com

HB: hbtmbelay652@gmail.com

RM: r.maddren@imperial.ac.uk

TL: t.landeryou@imperial.ac.uk

KS: s.kalahasti@imperial.ac.uk

AKO: alisonkate.ower@gmail.com

KM: kalnyou@yahoo.com

AEP: a.phillips05@imperial.ac.uk

UA: ufaysaaj@gmail.com

TE: tenderias@yahoo.com

AT: adugnatamiru@gmail.com

BG: bokregidey@yahoo.com

ZM: zmehari@ciff.org

BM: BMengistu@ciff.org

GTo: getachewtollera@gmail.com

GTa: getas73@yahoo.com

Abstract

Introduction: The goal of the Geshiyaro project is to break transmission of soil-transmitted helminthiasis and schistosomiasis in the Wolaita Zone of Ethiopia through preventive chemotherapy with albendazole (ALB) and praziquantel (PZQ), with the goal of 90% treatment coverage. To compare reported administrative coverage, and ensure that the Geshiyaro threshold

had been met, post-treatment coverage evaluation surveys (CES) were conducted to monitor the success of each round.

Methods: A community-based cross-sectional study was conducted in 13 woredas (districts) of the Wolaita Zone. All eligible individuals from the selected households were invited for an interview. The study design, sample size, analysis and report writing was done according to the World Health Organization CES guidelines for preventive chemotherapy.

Results: The study interviewed a total of 3,568 households and 18,875 individuals across 13 woredas in the Wolaita Zone. Overall, the survey coverage across all studied woredas was 81.5% (95% CI; 80.9 – 82.0) for both ALB and PZQ. Reported administrative coverage across all studied woredas was higher than survey coverage, 92.7% and 91.2% for ALB and PZQ respectively. Significant portion of the beneficiaries (17.6%) were not offered the drugs, and the main reason for not achieving the target coverage included beneficiaries absenteeism during Mass Drug Administration (MDA) (6.6% ALB, 6.8% PZQ), drug distributors failed to go to all households (4.7% ALB, 4.8% PZQ), and the beneficiaries unable to hear about the program (1.3% ALB, 1.7% PZQ).

Conclusion: It is important to take programmatic actions during the next MDA campaign to achieve the minimum Geshiyaro coverage threshold. Adequate training and supervision on reporting and tallying should be given to the drug distributers, supervisors and staff of the Ministry of Health (MoH). It is important to strengthen the social mobilization, motivation of drug distributers, and enhancing the engagement of MoH staff during the future MDA campaign.

Introduction

Neglected tropical diseases (NTDs) are a diverse group of infectious diseases which are among the most common diseases and affect one billion people globally (1-3). Among NTDs, soil-transmitted helminthiasis (STH) and schistosomiasis (SCH) are common and disproportionally affect those that live in poverty (4, 5). Ethiopia has the highest prevalence of both STH and SCH and it is estimated that 37.3 million and 79 million people live in SCH and STH endemic areas respectively (6, 7). The World Health Organization (WHO) recommends preventive chemotherapy treatment (PCT) with albendazole (ALB) and praziquantel (PZQ) as one of the interventions used to control and eliminate STH and SCH, respectively (8-10). Preventive chemotherapy distributed through mass drug administration (MDA), is used to treat at-risk populations at appropriate intervals (11, 12). In order to monitor the success of each round of the MDA, it is imperative to know the coverage and compliance of the PCT distribution that is, how many people in need of treatment swallowed the targeted drugs (12, 13). Coverage is the fraction or percentage of people out of the eligible population, and compliance describes how much of those offered swallowed the target drug (13). Without reliable information about PCT coverage it is a challenge to evaluate programme performance effectively, or indeed predict how the prevalence of infection and associated disease is impacted by the MDA (13).

Reported administrative PCT coverage data is determined using the reported coverage rate, which is calculated by aggregating the drug distributor's records and dividing by the estimated eligible population. Though the reported administrative coverage is still important for programme monitoring, it is prone to errors resulting from incorrect estimates of the target population, weak health information systems, underreporting, and intentional inflation of the number of those treated (13). Therefore, it is important to compare the reported coverage rates, using treatment coverage

evaluation surveys (CES) which are valuable tool for evaluating program performance (13). Coverage evaluation surveys are population-based surveys designed to provide precise statistical estimate of the PCT coverage that overcome many of the biases and errors that can undermine reported administrative coverage (13).

The Geshiyaro project is designed to break transmission of STH and SCH in the Wolaita Zone of Ethiopia through MDA and provision of water, hygiene and sanitation (WaSH) facilities. MDA and WaSH activities are organized and overseen by Ministry of Health (MoH) and the World Vision respectively. The Health Extension Workers (HEW) were used to implement the MDA campaign (14). Since 2018, as part of the Geshiyaro project, the whole eligible community members of Wolaita Zone started to take a bi-annual ALB and an annual PZQ treatment, with the goal of reaching a 90% treatment coverage at each round of the MDA. In 2021, a total of 22 woredas in the Zone implemented a community-wide MDA program for all eligible community members. Therefore, the study aimed to estimate the survey coverage and compare it with the reported administrative coverage and the Geshiyaro coverage threshold. This study also assessed reasons for not achieving the desired coverage target. The findings of the study will be important for the national program to improve future MDA campaigns to be implemented as part of the Geshiyaro project.

Methods

Study settings and period

This post-treatment CES was conducted in February 2021 in a random sample of population that received treatment in 13 woredas of the Wolaita Zone. The Zone is located in South West of the Southern Nations and Nationalities Peoples Region (SNNPR), 330Km from the capital, Addis

Ababa, Ethiopia. According to the recent government restructure, the Zone has a total of 22 woredas. As part of the Geshiyaro project, where the detailed protocol is published elsewhere (14), all eligible community members residing in the Zone took ALB and PZQ treatment in different time period. In this study, thirteen woredas that took both drugs in January 2021 were considered.

Study design

A community-based cross-sectional study design was used for the current study.

Sampling

This CES design used probability proportional to estimated size (PPES) to select enumeration areas (EAs), the lowest administrative unit, from 13 survey woredas. From each woreda, 30 EAs were selected randomly with probability proportional to the number of segments in the EAs, where a segment represents a group of roughly 50 households (HHs) (13). The Coverage Survey Builder (CSB), an excel-based tool, which is recommended by the WHO (13), was used to select the EAs from each woreda. Following the selection of the EAs, a segment was selected randomly from within each selected EA. Households to be included in the study were also randomly selected from the selected segment. The sampling interval (which is the interval between two selected HHs), automatically generated by the CSB, was used to determine which HHs in the segment were to be sampled to reach the expected sample size. Finally, all eligible household members living in the selected HH were interviewed. Exhaustive list of EAs with maps and the estimated number of HHs for the respective woredas in Wolaita Zone was obtained from the Ethiopian Central Statistics Authority (CSA). The latest population-based mini-survey in Ethiopia was done in 2019 (15). Figure 1 shows the summary of sampling scheme used to select study participants.

Figure 1: Sampling scheme at different stages, adapted from the WHO (13)

Study population

According to the Geshiyaro MDA program, ALB and PZQ had been provided to all individuals (≥ 1 and >4 years old, respectively) residing in the Wolaita Zone. Therefore, the study population consists of all community members residing in 13 woredas who are eligible for ALB and PZQ drugs. Preventive chemotherapy survey coverage was estimated from the eligible study population which was considered as the denominator for the calculation of survey coverage.

Sample size calculations

The sample size was determined automatically using the WHO excel-based CSB (13) by assuming 0.05 margin of error, a 95% confidence interval (CI), non-response rate of 15%, and a design effect of 4 with an expected coverage rate that was obtained from the reported administrative report of drug distributers (HEWs) during the MDA. The expected administrative coverage along with the detailed parameter used to calculate the sample size for each woreda can be seen in supporting information 1.

Data collection

Data was collected by well-trained health professionals using the Survey CTO application installed in each data collector's mobile phones. The adopted questionnaire from the WHO CES tool (13) was used to capture pertinent information from the participants (Supporting information

2). The data collectors along with the local guides identified a walking route that passed by every house in the selected segment. Pertinent information was obtained from all eligible household members living in the selected HH. Information for young children (<10 years) were collected from their primary caretakers. There was a re-visit by the data collectors if there was no one available at the selected HH during the time of interview. The data collectors interviewed another adult on behalf of the absent member of the HH if the individual is still absent during a re-visit. The data collectors, as per the protocol, considered the next HH if the selected HH was a business center. Interview continued until the required sample size was obtained in each segment.

Data quality control

To maintain the quality of the collected data, four days of intensive training was given for data collectors and supervisors regarding; introduction to the Geshiyaro project, Survey CTO application usage and the details on the survey methods. The WHO CES tool was adopted for the current study which avoided potential measurement errors. Additionally, the recommended WHO coverage evaluation protocol for preventive chemotherapy was adapted for methodology and to generate a report (13). A daily data quality management and assurance report was generated and communicated to the field team by the data manager which was used to correct any errors, according to the protocol and standard while on the field. In order to avoid recall bias, data collection was done within one month of the MDA campaign dates. Additionally, the survey team presented sample drug pills for the respondents to help them recall respective drugs. Furthermore, to avoid social desirability bias, the HEWs who participated in drug distribution were not used as a local guide in this CES.

Data Analysis

Two percentage measures were calculated in this study*:* the ‘self- reported survey coverage’, of what percentage of the eligible population swallowed a pill (which is used to compare with the administrative reported coverage) and the ‘program reach’ (which tell us whether the individual is offered the drug or not). Individual compliance with the MDA treatment was identified by comparing the survey coverage to the programme reach. Self-reported survey coverage was compared with the MoH reported administrative coverage and the target Geshiyaro coverage threshold. The following two formulas were used to calculate the survey coverage and program reach respectively.

$$\text{Surveyed coverage} = \frac{Number\ of\ individuals\ who\ \boldsymbol{swallowed}\ the\ drug}{Total\ number\ of\ individuals\ surveyed}$$

$$\text{Program reach} = \frac{Number\ of\ '\text{yes}'\ responses\ to\ having\ been\ \boldsymbol{offered}\ the\ drug}{Total\ number\ of\ individuals\ surveyed}$$

The WHO CSB “Results Entry Form” which is available in CSB excel sheet (13) was used to get estimated survey and program reach coverages. The 95% CI around the survey coverage was also automatically calculated using the CSB analysis tool.

Ethics statement

The study was approved by Institutional Review Board (IRB) at the Scientific and Ethical Review Office of the Ethiopian Public Health Institute. Letter of support and explanation about the purpose of the study was provided to all concerned bodies. Before conducting any interview, verbal consent was taken from the head of the household or an adult after providing all necessary information about the study in the local language that every participant can understand. Assent

for young children (<10 years) was obtained from their primary guardian. The confidentiality of all the participants was strictly kept.

Results

Total number of households and individuals interviewed

As seen in Table 1, a total of 3,568 HHs and 18,875 individuals were interviewed across the 13 studied woredas in Wolaita Zone of Ethiopia. The distribution of study participants across sex, was even. Relatively speaking, school-aged children (SAC) (age: 5 to14) and pre-school aged children (PSAC) (age: 1 to 4) had high and low sample representation respectively.

Table 1: Distribution of interviewed HHs and individuals across each woreda, Wolaita Zone, February 2021

**Woreda**	**Total HHs interviewed**	**Total individuals interviewed**	**Female**	**Male**	**1 to 4**	**5 to 14**	**15 to 20**	**21 to 35**	**35+**
Boloso Sore	251	1,391	663	728	142	449	238	332	230
Sodo Town	272	1,415	726	689	96	361	274	404	280
Diguna Fango	269	1,445	707	738	129	440	255	362	259
Abala Abaya	314	1,701	833	868	158	576	305	347	315
Kindo Koysha	288	1,652	851	801	125	530	300	431	266
Offa	268	1,432	727	705	150	437	216	299	330
Sodo Zuria	273	1,398	700	698	105	429	250	287	327
Tebela Town	270	1,364	687	677	108	346	287	364	259
Humbo Woreda	279	1,525	738	787	115	512	259	311	328
Hobicha	269	1,543	761	782	124	518	287	312	302

Bayra Koysha	276	1,260	639	621	101	375	182	295	307
Kawo Koysha	273	1,401	718	683	137	462	201	262	339
Gesuba Town	266	1,348	674	674	90	372	271	320	295
**Total**	**3,568**	**18,875**	**9,424**	**9,451**	**1,580**	**5,807**	**3,325**	**4,326**	**3,837**

Survey coverage of albendazole and praziquantel by woreda

The overall survey coverage among the eligible population in the studied woredas was 81.5% (95% CI; 80.9 – 82.0) for both ALB and PZQ. The lowest survey coverage for both ALB and PZQ were observed in Sodo town, at 52.3% and 51.3% respectively. On the other hand, high survey coverage was reported in Abala Abaya, Sodo Zuria and Bayra Koysha woredas. Five of thirteen woredas had survey coverage above 85% for both drugs (Table 2 and 3).

The overall survey coverage among male and female for ALB were 81.8% and 81.1%, while PZQ were 81.1% and 81.9% respectively. Generally, the overall survey coverage of both drugs among males and females were not statistically different ($p=0.9$ for both drugs). The highest survey coverage for both drugs was observed in school-age children at 87.6% for ALB and 86.4% for PZQ. In contrast, pre-school age children had the lowest survey coverage for ALB (76.1%), and individuals within age 21 to 35 had the lowest survey coverage for PZQ (76.3%).

The reported administrative coverage for all studied woredas in the 2021 Geshiyaro MDA campaign was greater than the survey coverage. The highest discrepancy between the two figures was observed in Sodo, Tebela and Gesuba towns. The discrepancy in survey and reported administrative coverage was lower in five of thirteen woredas for ALB and seven of thirteen for PZQ. There were no woreda who met the Geshiyaro coverage threshold, however, four of thirteen woredas had a survey coverage near to the threshold (Table 2 and 3).

Table 2: Comparison of survey coverages with reported administrative coverages, program reach and the Geshiyaro threshold (which is ≥90%) for ALB, Wolaita Zone, February 2021

Woreda	Reported coverage (%)	Survey coverage with 95% CI	Program reach	Geshiyaro threshold	Drug acceptance (%)
Boloso Sore	93.6	81.9 (74.7, 87.5)	82.8	90	99.1
Sodo Town	83.5	52.3 (44.7, 59.8)	53.2	90	98.3
Diguna Fango	97.4	83.8 (78.3, 88.1)	84.7	90	98.9
Abala Abaya	83.1	88.2 (84.0,91.4)	88.9	90	99.2
Kindo Koysha	93.7	84.0 (79.9, 87.4)	84.4	90	99.0
Offa	92.0	85.4 (80.0, 89.5)	85.6	90	99.9
Sodo Zuria	93.8	88.4 (84.7, 91.3)	89.2	90	99.1
Tebela Town	93.7	75.3 (69.4, 80.4)	78.5	90	95.9
Humbo Woreda	93.2	87.02 (82.4, 90.6)	88.5	90	98.3
Hobicha	90.2	78.8 (69.5, 85.9)	79.7	90	98.9
Bayra Koysha	97.3	89.1 (84.7, 92.4)	89.3	90	99.8
Kawo Koysha	95.2	83.9 (77.3, 88.9)	84.9	90	98.8
Gesuba Town	98.0	79.6 (74.8, 83.7)	80.04	90	99.4
**Total**	**92.7**	**81.5 (80.9-82.0)**	**82.4**	**90**	**98.8**

Table 3: Comparison of survey coverages with reported administrative coverages, program reach and the Geshiyaro threshold for PZQ, Wolaita Zone, February 2021

Wereda	Reported coverage (%)	Survey coverage with 95% CI	Program reach	Geshiyaro threshold	Drug acceptance (%)
Boloso Sore	92.2	83.5 (75.9, 89.0)	84.3	90	99.1

Sodo Town	76.6	51.3 (43.7, 58.9)	52.2	90	98.3
Diguna Fango	92.7	82.6 (77.0, 87.1)	83.5	90	98.9
Abala Abaya	82.2	88.2 (84.0, 91.4)	89.5	90	98.6
Kindo Koysha	92.4	84.8 (80.6, 88.2)	85.1	90	99.7
Offa	92.8	85.9 (80.4, 90.0)	86.2	90	99.9
Sodo Zuria	93.6	88.1 (83.7, 91.4)	88.9	90	99.0
Tebela Town	92.1	74.3 (68.4, 79.8)	77.9	90	95.6
Humbo Woreda	92.7	86.7 (82.3, 90.1)	87.2	90	99.4
Hobicha	91.7	80.2 (70.9, 87.1)	80.9	90	99.0
Bayra Koysha	96.0	89.0 (84.6, 92.3)	89.1	90	99.9
Kawo Koysha	94.4	84.3 (77.7, 89.2)	85.6	90	98.4
Gesuba Town	96.1	79.7 (74.9, 83.7)	80.1	90	99.5
**Total**	**91.2**	**81.5 (80.9 – 82.0)**	**82.4**	**90**	**98.9**

Reasons for not being offered albendazole and praziquantel

The overall program reach among the eligible population for both drugs was 82.4% (95% CI; 81.8 – 82.9). This indicates that a significant portion of the beneficiaries were not offered the drugs during the time of the MDA campaign. The main reason for not achieving the target coverage included participant absenteeism during MDA (6.6% ALB and 6.8% PZQ), drug distributors (HEWs) failed to go to all households (4.7% ALB and 4.8% PZQ), and the beneficiaries unable to hear about the program (1.3% ALB and 1.7% PZQ) (Table 4). The lowest program reach for both ALB and PZQ were observed in Sodo town, at 53.2% and 52.2% respectively. Abala Abaya, Sodo Zuria, Humbo and Bayra Koysha reported a higher program reach for both drugs. The highest program reach for both ALB and PZQ were observed in school-age children at 88.4% and 87.4%

respectively. Relatively speaking, individuals within age 21 to 35 had the lowest program reach for both ALB (77.2%) and PZQ (76.9%). Overall, the program reach among males and females were not statistically different (*p=0.9* for both drugs)

The overall treatment acceptance (ratio of those who swallowed the drugs amongst those who have been offered drugs) was high for both drugs. Only 1.2 % and 1.1% of those offered reported not taking ALB and PZQ respectively.

Table 4: Reasons for not being offered the drugs, Wolaita Zone, February 2021

Reason not offered drugs	ALB, n (%)	PZQ, n (%)
Underage	164 (0.87)	42 (0.24)
Pregnant	247 (1.31)	251 (1.45)
Breastfeeding	17 (0.09)	15 (0.09)
Too sick	145 (0.77)	139 (0.80)
Absent	1254 (6.64)	1180 (6.82)
Not heard about programme	252 (1.33)	287 (1.66)
Drugs finished/ran out	40 (0.21)	19 (0.11)
HEW did not come	887 (4.69)	825 (4.77)
Other	150 (0.79)	133 (0.77)

Discussions

This coverage evaluation survey was used to estimate the survey coverage and compared it with the reported administrative coverage and the target Geshiyaro coverage threshold. Additionally,

reasons for not achieving the desired coverage target were identified. It was done as part of the different evaluation activities being implemented by the ongoing Geshiyaro project (14).

Overall survey coverage across all studied woredas was 81.5% for both ALB and PZQ. The reported administrative coverage across all studied woredas was greater than the survey coverage; where majority of the woredas reported above 90% coverage. The highest discrepancy between the two figures were observed in Sodo, Tebela and Gesuba towns. This is an indication that there was a problem with reporting system employed by the drug distributors during the MDA campaign. The drug distributors were wrongly reporting on ingestion of the drugs. Prior to the next round of MDA, it is important for the MoH to do a data quality self-assessment to isolate problems related to the reporting system. Adequate training and supervision on reporting and tallying should be given to the drug distributors, supervisors and MoH staff prior to the next round of the MDA. It is also important to motivate the drug distributors to have a better reporting system. MoH also needs to check if there is any issue related to the denominator used to calculate the reported administrative coverage in these woredas (the eligible population given by the government census bureau). Discrepancy between the reported administrative and survey coverage was also seen on other similar study in Ethiopia (16).

The discrepancy in survey and reported administrative coverage was lower in five of thirteen woredas for ALB and seven of thirteen for PZQ. According to the WHO, the estimates obtained

from these two figures are considered to have a lower discrepancy or are similar if the reported administrative coverage lies either within the 95% CI around the value obtained in the survey coverage or is within +/- 10 percentage points of the survey coverage (13). (Detailed validation interpretation adapted from the WHO coverage evaluation survey can be seen from supporting information 3). This implies that, there was no issue with the reporting system in these woredas where a lower discrepancy in survey and reported administrative coverage were observed. Therefore, it is important to encourage the drug distributors and supervisors working in these woredas to continue with their current reporting practices during the next rounds of the MDA.

In all the surveyed woredas, the survey coverage is below the Geshiyaro coverage threshold (≥90% coverage) for both ALB and PZQ. Therefore, it is important to strengthen the MDA campaign further in all of the studied woredas during the next rounds of the MDA to achieve the minimum Geshiyaro coverage threshold. It is important to note that the survey coverage was below 80% in Sodo town (52.3% ALB and 51.3% PZQ), Gesuba town (79.6% ALB and 79.7% PZQ) and Tebela town (75.3% ALB and 74.5% PZQ). The survey coverage for Sodo town is far below the Geshiyaro target coverage by 38%. This implies that the MDA program is not functioning well in these towns. Relative to the rural woredas, urban towns still need great attention, and if programmatic actions will not be taken during the next rounds of the MDA, it might create a bottle neck for STH/SCH transmission break in the Wolaita Zone.

Program reach is classified as the individual is offered the drug or no, irrespective of swallowing. It measures if there is any issue with the supply chain of the drug, performance of the drug distributors and allows the program to highlight areas where there needs additional attention (13). The overall program reach among the eligible population was 82.4% for both ALB and PZQ. This indicates that there were considerable number of beneficiaries who were not offered these drugs during the MDA campaign. The main reason for not achieving the target coverage included beneficiaries' absenteeism during MDA, drug distributors failed to go to all households, and the beneficiaries unable to hear about the program. Therefore, during next rounds of the MDA, it is important to address reasons for not being offered the drugs which included strengthening the social mobilization, repeated visit by the drug distributors, motivation of the drug distributors, and enhancing the engagement of MoH staff during the future MDA campaign.

The overall treatment acceptance (ratio of those who swallowed the drugs amongst those who have been offered the drugs) is high for both drugs. Therefore, it is important to continue with the current available information and education campaigns in all studied woredas.

Conclusions

It is important to take programmatic actions during the next MDA campaign to achieve the minimum Geshiyaro coverage threshold. Adequate training and supervision on reporting and tallying should be given to the drug distributers, supervisors and MoH staff to improve upon the data capture system currently in place. It is important to strengthen the social mobilization,

motivation of drug distributers, and enhancing the engagement of MoH staff during the future MDA campaign.

Acknowledgements

We are very much grateful for all individuals who participated and provided their information for the study. We are indebted to all administrators at each level who facilitated the implementation of the current study.

References

1. Herricks JR, Hotez PJ, Wanga V, Coffeng LE, Haagsma JA, Basanez MG, et al. The global burden of disease study 2013: What does it mean for the NTDs? PLoS Negl Trop Dis. 2017;11(8):e0005424.
2. WHO. Working to overcome the global impact of neglected tropical diseases: first WHO report on neglected tropical diseases. Geneva: World Health Organization; 2010.
3. WHO. Ending the neglect to attain the sustainable development goals: a road map for neglected tropical diseases 2021–2030: overview. Geneva: World Health Organization; 2020 2020. Report No.: 9789240018792 (electronic version)

4. Pullan RL, Smith JL, Jasrasaria R, Brooker SJ. Global numbers of infection and disease burden of soil transmitted helminth infections in 2010. Parasit Vectors. 2014;7:37.
5. van der Werf MJ, de Vlas SJ, Brooker S, Looman CW, Nagelkerke NJ, Habbema JD, et al. Quantification of clinical morbidity associated with schistosome infection in sub-Saharan Africa. Acta Trop. 2003;86(2-3):125-39.
6. Negussu N, Mengistu B, Kebede B, Deribe K, Ejigu E, Tadesse G, et al. Ethiopia Schistosomiasis and Soil-Transmitted Helminthes Control Programme: Progress and Prospects. Ethiop Med J. 2017;55(Suppl 1):75-80.
7. Maddren R, Phillips A, Ower A, Landeryou T, Mengistu B, Anjulo U, et al. Soil-transmitted helminths and schistosome infections in Ethiopia: a systematic review of progress in their control over the past 20 years. Parasites & Vectors. 2021;14(1):97.
8. WHO. Accelerating work to overcome the global impact of neglected tropical diseases – A roadmap for implementation. WHO; 2012.
9. WHO. Soil-transmitted helminthiases : eliminating as public health problem soil-transmitted helminthiases in children : progress report 2001-2010 and strategic plan 2011-2020. Geneva: World Health Organization; 2012.

10. Mupfasoni D, Bangert M, Mikhailov A, Marocco C, Montresor A. Sustained preventive chemotherapy for soil-transmitted helminthiases leads to reduction in prevalence and anthelminthic tablets required. Infect Dis Poverty. 2019;8(1):82.
11. WHO. Preventive chemotherapy in human helminthiasis. 2006.
12. Webster JP, Molyneux DH, Hotez PJ, Fenwick A. The contribution of mass drug administration to global health: past, present and future. Philos Trans R Soc Lond B Biol Sci. 2014;369(1645):20130434.
13. WHO. Preventive chemotherapy: tools for improving the quality of reported data and information: a field manual for implementation. Geneva: World Health Organization; 2019 2019.
14. Mekete K, Ower A, Dunn J, Sime H, Tadesse G, Abate E, et al. The Geshiyaro Project: a study protocol for developing a scalable model of interventions for moving towards the interruption of the transmission of soil-transmitted helminths and schistosome infections in the Wolaita zone of Ethiopia. Parasites & Vectors. 2019;12(1):503.
15. Ethiopian Public Health Institute - EPHI, Federal Ministry of Health - FMoH, ICF. Ethiopia Mini Demographic and Health Survey 2019. Addis Ababa, Ethiopia: EPHI/FMoH/ICF; 2021.
16. Asfaw MA, Zerdo Z, Churko C, Seife F, Yihune M, Chisha Y, et al. Preventive chemotherapy coverage against soil-transmitted helminth infection among school age children: Implications from coverage validation survey in Ethiopia, 2019. PloS one. 2020;15(6):e0235281-e.

Supporting information

S1 Appendix: Validation interpretation, adapted from WHO coverage evaluation survey (13)

S2 Appendix: Household questionnaire, adapted from WHO coverage evaluation survey (13)

S3 Appendix: Parameter values used for sample size calculation

Figure

Click here to access/download;Figure;Fig 1.tif

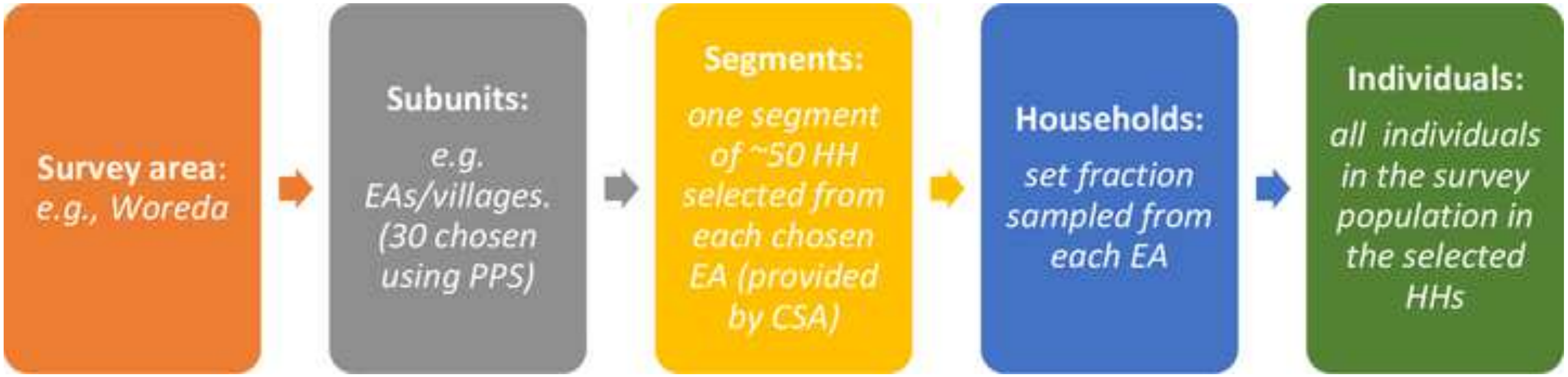

Click here to access/download
Supporting Information
Supporting information.docx
