# Supplementary material for: Coverage evaluation surveys following soil-transmitted helminthiasis and schistosomiasis mass drug administration in Wolaita Zone of Ethiopia—The Geshiyaro project
Source: PLoS One. 2021 Dec 21;16(12):e0260722. doi: 10.1371/journal.pone.0260722 (PMC8691621; doi:10.1371/journal.pone.0260722)
Supplement: Attachment — Submitted filename: Reveiw point.docx [file pone.0260722.s004.docx]

**Comments to the Author**

The manuscript describes Coverage evaluation surveys following soil-transmitted helminthiasis and schistosomiasis mass drug administration in Wolaita Zone of Ethiopia

Comment

- What is the eligible population for PC PZQ and ALB in the survey site and what platform according to WHO , please add more information in the introduction?
- In introduction Please add information regarding the PZQ and ALB dose administered. For PZQ please also describe the dose base on age or ?
- how many structure questions have been asked of the community ? is the any question related to adverse event?
- Line 200 Table 1., Please grouping as age group🡪 1-4,5-14, 15-20,21-34, 35+
- Figure 1 ( from WHO source and you need permission ). Please add new figure which adaptable for the survey site ( including EAs, subdunit, HH etc)
- The surveyed coverage is 52.3% and the reported coverage is 83.5% ( the coverage compliance gap is high) please more explore in discussion chapter.
- In line 256 “The drug distributors were wrongly reporting on ingestion of the drugs” please change this word because author do not have evidence about this. The reason can be the drug distributor only deliver and no mandatory to ingested immediately (author should add the similar reference)
- In discussion, please elaborate what the interpretation of if reported coverage and survey coverage lower, higher or similar, in other word the converting the results into programmatic action
- The conclusion sections of the abstract and manuscript should not be the same.
- Grammatical error in some parts is needed, author may consult Professional English for editing
