# Supplementary material for: Coverage evaluation surveys following soil-transmitted helminthiasis and schistosomiasis mass drug administration in Wolaita Zone of Ethiopia—The Geshiyaro project
Source: PLoS One. 2021 Dec 21;16(12):e0260722. doi: 10.1371/journal.pone.0260722 (PMC8691621; doi:10.1371/journal.pone.0260722)
Supplement: Attachment — Submitted filename: Minor points rev.docx [file pone.0260722.s005.docx]

Overall, it looks good now, but some minor changes are required.

Point number 4 : Line 200 Table 1., Please grouping as age group◊ 1-4,5-14, 15-20,21-34, 35+

**Not as I expected** Please add **Age group** at colum above age (see below)

Line 92 Please revised the sentences ~~Mothers in their third trimester are advised~~

~~93 against taking ALB or PZQ~~ to ….Mothers in their third trimester are advised **not to take** ALB or PZQ
