# Supplementary material for: Coverage evaluation surveys following soil-transmitted helminthiasis and schistosomiasis mass drug administration in Wolaita Zone of Ethiopia—The Geshiyaro project
Source: PLoS One. 2021 Dec 21;16(12):e0260722. doi: 10.1371/journal.pone.0260722 (PMC8691621; doi:10.1371/journal.pone.0260722)
Supplement: Attachment — Submitted filename: Response to Reviewers.docx [file pone.0260722.s006.docx]

To Mariya Y Pakharukova
Academic Editor
PLOS ONE

Subject: Submission of revised version of a manuscript that addresses the points raised during the review process
Manuscript Number: PONE-D-21-27754R2

Dear Mariya Y Pakharukova and reviewer,
Thank you so much for reviewing our revised manuscript entitled “Coverage evaluation surveys following soil-transmitted helminthiasis and schistosomiasis mass drug administration in Wolaita Zone of Ethiopia- The Geshiyaro project”. Please find below point-by-point responses to the few comments made by the Academic Editor and the second reviewer.

A. **Academic Editor**

| **S.no** | **Questions raised** | **Responses made** |
| --- | --- | --- |
| 1. | Please review your reference list to ensure that it is complete and correct. If you have cited papers that have been retracted, please include the rationale for doing so in the manuscript text, or remove these references and replace them with relevant current references. Any changes to the reference list should be mentioned in the rebuttal letter that accompanies your revised manuscript. | - Thank you so much. We have used a Reference Manger to cite the references. We haven't used retracted articles in our manuscript. We have made some modification on reference number 11 (WHO. Preventive chemotherapy in human helminthiasis. 2006). This reference has now been changed in the following way in the revised manuscript ( page 19, lines 351-353).   Crompton, David WT, WHO. Preventive chemotherapy in human helminthiasis : coordinated use of anthelminthic drugs in control interventions : a manual for health professionals and programme managers. Geneva: World Health Organization; 2006.   - We have also deleted reference # 15 (WHO. Preventive chemotherapy in human helminthiasis : coordinated use of anthelminthic drugs in control interventions : a manual for health professionals and programme managers. Geneva: World Health Organization; 2006) as it is similar to reference # 11. |

B. **Reviewers' comments**

| **Reviewer # 2** | | |
| --- | --- | --- |
| **S.no** | **Questions raised** | **Responses made** |
| 1. | Point number 4 : Line 200 Table 1., Please grouping as age group 1-4,5-14, 15-20,21-34, 35+. Please add Age group at column above age. | - Thank you so much and this has been corrected per the reviewer’s suggestion (page 11, line 211). |
| 2. | Line 92 Please revised the sentences Mothers in their third trimester are advised 93 against taking ALB or PZQ to ….Mothers in their third trimester are advised not to take ALB or PZQ | - We accept the comment and revision on these sentences has been made in the revised manuscript (page 5, lines 92-93). |
